# Soft Gelatin Films Modified with Cellulose Acetate Phthalate Pseudolatex Dispersion—Structure and Permeability

**DOI:** 10.3390/polym10090981

**Published:** 2018-09-03

**Authors:** Bartosz Maciejewski, Anna Ström, Anette Larsson, Małgorzata Sznitowska

**Affiliations:** 1 Department of Pharmaceutical Technology, Medical University of Gdańsk, Hallera 107, 80-416 Gdańsk, Poland; b.maciejewski@gumed.edu.pl; 2 Pharmaceutical Technology, Department of Chemistry and Chemical Engineering, Chalmers University of Technology, SE-412 96 Gothenburg, Sweden; anna.strom@chalmers.se (A.S.); anette.larsson@chalmers.se (A.L.); 3 SuMo BIOMATERIALS, VINN Excellence Center, SE-412 96 Gothenburg, Sweden

**Keywords:** gelatin, gastro-resistant capsule, coacervation, microscopy, permeability, polyelectrolytes

## Abstract

Gastroresistant material, based on gelatin and intended to form capsule shells, was characterized. The films were obtained by mixing a gelatin solution with cellulose acetate phthalate (CAP) pseudolatex at an elevated temperature. Microscopic and spectroscopic analyses of the films—intact or subjected to the acidic treatment—were performed, along with a permeability study of tritium-labeled water. A uniform porous structure formed by CAP within the gelatin gel was observed. The results demonstrated that no interaction of a chemical nature occurred between the components. Additionally, the performed permeability and solubility studies proved that the diffusion of water through the membranes at an acidic pH can be noticeably reduced by adding carrageenan as a secondary gelling/thickening agent.

## 1. Introduction

Utilizing gelatin in a variety of applications has attracted a great deal of interest recently. Due to many characteristic properties, gelatin can be considered for use in fields such as food, food packaging, biomaterials and pharmaceutical technology. In the latter, gelatin is studied for its suitability in controlled drug delivery systems (microcapsules, microspheres) and film formulations, such as oral thin films and pharmaceutical capsules [1,2,3,4,5].

Gelatin is a natural biopolymer obtained through the partial hydrolysis of collagen derived from animals. The structure of gelatin allows for the organization of random coils into helices and turns, under the influence of changing temperatures, resulting in thermo-reversible gelling behavior [2,6,7,8]. Along with the property of creating highly elastic gels in a wide range of concentrations at room temperature, gelatin also has good film-forming capabilities. Gelatin and gelatin films are soluble in water at temperatures of ca. 35 °C, while in cold water swelling and softening can be observed [9]. The solubility of gelatin is not affected by the pH of the medium.

Gelatin films are traditionally used as a capsule shell material [10]. Despite numerous gelatin substitutes, which are gaining popularity in hard capsule technology, it is difficult to find a substitute material that allows for the production of soft gelatin capsules with a popular rotary die method.

As with its substitutes, there are also difficulties in assuring the modified release of active substances from soft capsules. Coating soft capsules is a rarely used method to assure the gastro-resistance of soft capsules. This process brings numerous technological difficulties, such as sticking capsules together, cracking and poor adhesiveness of the coating film [11,12]. Therefore, developing an acid-insoluble material suitable for producing capsule shells and allowing for the manufacture of gastro-resistant soft capsules is a challenge. In scientific and patent literature, records of attempts to incorporate acid-insoluble polymers into gelatin gel prior to encapsulation have been present since the 1940s [13,14]. However, it was only after the year 2000 that a description of the material for soft capsules appeared, with gelatin being used in a mixture with well-known acid-insoluble polymers such as methacrylic acid copolymers, hypromellose phthalate, hypromellose acetate succinate, cellulose acetate phthalate, etc., and mixtures thereof. It is noteworthy that the aforementioned polymer blends need to contain an alkalizing agent, such as NaOH, NH_4_OH or triethanolamine, in order to dissolve functional polymers into clear solutions [15]. Only in one recent patent application has the optional use of alkalizing substances been proposed [16]: The authors explain that in non-alkalized films, a higher amount of available non-ionized carboxylic acid groups contributes to a higher resistance of the blend to low pH media.

In our previous work, gelatin-based films that do not disintegrate at a pH ≤ 4.5 for at least 2 h were described. On the other hand, fast disintegration/dissolution, within 10 min or 15 min, was observed in buffers at a pH of 5.5–6.8, and in less than 10 min in biorelevant media: FaSSIF and FeSSIF (Fasted or Fed State Simulated Intestinal Fluid) [17]. The films were prepared by mixing gelatins at certain conditions with a commercial aqueous dispersion of cellulose acetate phthalate (Aquacoat CPD^®^, FMC Biopolymer, Philadelphia, PA, USA) without the addition of any alkalizing substances. Aquacoat CPD^®^ is a commercial cellulose acetate phthalate (CAP) dispersion widely used for the gastro-resistant coating of various solid drug forms. Using this product in the following investigation instead of a pure CAP allowed for the easier formulation of modified gelatin-based films, and made the concept more attractive to the pharmaceutical industry.

The aim of the present study was to determine possible mechanisms leading to the formation of Aquacoat-modified gelatin films resistant to disintegration in simulated gastric conditions. For this purpose, microscopic methods and ATR-FTIR spectroscopy were used, while a radio-labeled water permeability measurement was employed to characterize the barrier properties of the films in the acidic environment (pH 1.2).

## 2. Materials and Methods

### 2.1. Materials

Bovine gelatin (type B) from Italgelatine (Cuneo, Italy) was generously provided by Curtis Health Caps (Wysogotowo, Poland), and Aquacoat CPD^®^ (FMC Biopolymer, Philadelphia, PA, USA), a 30% aqueous pseudolatex dispersion of cellulose acetate phthalate (CAP), was a gift from IMCD Polska (Warsaw, Poland). Glycerol (99.5%) was purchased from Glackonchemie (Merseburg, Germany), ί-carrageenan was purchased from Sigma Aldrich (Saint Louis, MO, USA), and tritium-labeled water was purchased from Amersham (Little Chalfont, UK). The liquid scintillation cocktail (Ultima Gold^®^**™**) used in the permeability test was purchased from Perkin Elmer (Waltham, MA, USA).

### 2.2. Preparation of Films

The film compositions are shown in Table 1. The film-forming masses were prepared in a single vessel. The amount of glycerol in the film-forming solutions was the same in all the formulations, while the amount of Aquacoat solids varied from 10 to 30% by the total mass of polymers (Gelatin + Aquacoat).Aquacoat CPD consists of CAP (approx. 23%), poloxamer (6%), water (70%) and residues of free phthalic and acetic acids (around 1%). Therefore, in the final compositions, in addition to CAP particles, poloxamer can be found (with a final concentration in the film of approx. 1.4–4.1%). Hence, when referring to the “Aquacoat content”, the authors mean the total content of CAP and poloxamer in the film composition.

Aquacoat CPD, glycerol, gelatin and water were added to a round flask and mixed with paddle stirrer at 40 rpm while heating to 80 °C in a water bath for 2 h. After that, the mass was deareated under vacuum. Hot mass was casted on a glass plate using a TLC plate coater (Camag, Muttenz, Switzerland) and the thickness was aligned using a 1500 µm gap, which finally resulted in dry films with a thickness of ca. 450 µm (measured using an ElectroPhysik MiniTest 730 thickness analyzer (Cologne, Germany)). The films were dried for 90 min in an air dryer at room temperature (20–25 °C) and with a humidity of 40–60% RH and were then put into storage at room temperature in a dessicator(15–25% RH). The final moisture content measured thermogravimetrically was around 4% (WPS 210 S moisture analyzer, Radwag, Radom, Poland). In addition, reference films consisting of gelatin, glycerol and water, or made of pure Aquacoat CPD only, were prepared.

### 2.3. Scanning Electron Microscopy (SEM)

SEM photographs were taken with an Ultra 55 Scanning Electron Microscope (LEO Electron Microscopy, Cambridge, UK) using a field emission gun. The images were taken for the dry film surface, as well as for the freeze-dried films after immersion in 0.1 M HCl at 37 °C for 3 h (see paragraph 2.7).

### 2.4. Energy-Dispersive X-ray Spectroscopy (EDX)

EDX analyses were conducted using an Ultra 55 SEM (LEO Electron Microscopy Ltd., Cambridge, UK) equipped with an Inca X-Sight energy-dispersive X-ray detector (Oxford Instruments, Abingdon, UK). The gelatin film samples, non-modified (0% Aquacoat-GEL) and modified with 30% Aquacoat (GA1), were investigated. The distribution of nitrogen in both films was measured to distinguish the gelatin domains (containing nitrogen atoms) from the CAP domains (without nitrogen).

### 2.5. Confocal Laser Scanning Microscopy (CLSM)

The CLSM images were obtained using a TCS SP5 II confocal laser scanning microscope (Leica Microsystems, Wetzlar, Germany) with a 488 nm laser with a 50× 0.9 NA air objective and optical zoom. The samples were stained with acridine orange, which binds to the protein domains, thus revealing the pattern of the separated gelatin and CAP phases.

### 2.6. Attenuated Total Reflection-Fourier Transform Infra-Red (ATR-FTIR) Spectroscopy

The IR spectra of the films were obtained using a Frontier FT-IR Spectrometer (Perkin Elmer, Waltham, MA, USA) and a GladiATR sampling device with diamond ATR crystal (Pike Technologies, Madison, WI, USA). The measurements were made in a wavenumber range from 4000 to 400 cm^−1^ and the spectra are presented as % transmittance. Both surfaces of the film samples were tested. To better visualize the differences between the spectra, the peak intensity ratios of certain samples were calculated and showed in the graphs.

### 2.7. Swelling and Fraction Soluble in HCl

The swelling of the membranes was measured using the method described by Peh and Wong (1999) [18] with slight modifications. Prior to the test, the films were analyzed for moisture content. Samples of 1 × 1 cm in size were weighed and placed for 3 h in vials containing 20 mL of 0.1 M HCl at 37 °C, without shaking. After 15, 30, 45, 60, 90, 120, 150 and 180 min, the film samples were carefully removed, wiped of excessive fluids and the weight gain was determined. The experiments were carried out in triplicates.

The mass fraction of the membranes soluble in 0.1 M HCl was measured as a mass loss after the samples were dried in an air dryer at 105 °C.

### 2.8. Permeability Study

The permeability of the tritium-labeled water through the films at 37 °C was studied. The tested film sample was placed between the donor and the acceptor chambers of a vertical diffusion cell [19,20]. The permeation area was 1.77 cm^2^. Each compartment of the chamber was filled with 15.0 mL of the 0.1 M HCl, and 5 µL of radio-labeled water was added to the donor compartment. The test was conducted for 3 h and 0.5 mL samples were taken from the acceptor compartment every 15 min, replaced with 0.5 mL of fresh 0.1 M HCl. After that, 4 mL of the scintillation cocktail (Ultima Gold™) was added, and the radioactivity of the withdrawn samples was measured (as Decays Per Minute—DPM) using a liquid scintillation counter (Tri-Carb 2810 TR, Perkin Elmer, Waltham, MA, USA). The films were tested in duplicates. The permeability coefficient P was calculated using the following equations [21]:

(1) $$\;-\ln\frac{\left(c_{0}-2c_{t}\;\right)}{c_{0}}=P\frac{2\;A}{V}t$$

where *c*_0_ is the concentration of radioactive water in the donor cell at the time *t* = 0, *c_t_* is the concentration of radioactive water in the acceptor cell at the time *t*, *V* is the volume of the donor *V*_D_ or acceptor cell *V_A_* (in these experiments *V_D_* = *V_A_*) and *A* is the area of the film over which the radioactive water is transported.

Equation (1) can be solved for *P* by plotting the logarithm of the concentration gradient on the graph against time, and determining the slope factor (a) of the obtained line (according to the linear function equation *y* = a*x* + b):

(2) $$\;a=P\;\frac{2\;A\;}{V}$$

The slope stands for the diffusion rate of the permeating agent. The final equation used to calculate the permeability coefficient, corrected with the thickness of the film samples, is as follows:

(3) $$\;P=\frac{Flow*h*V\;}{2\;A}$$

where: *h* is thickness of the investigated samples, *V* is the chamber volume, and *A* is the permeation area of the film samples. The permeation coefficient was expressed in (cm^2^ s^−1^).

## 3. Results and Discussion

### 3.1. Microscopic Structure

**Figure 1:**
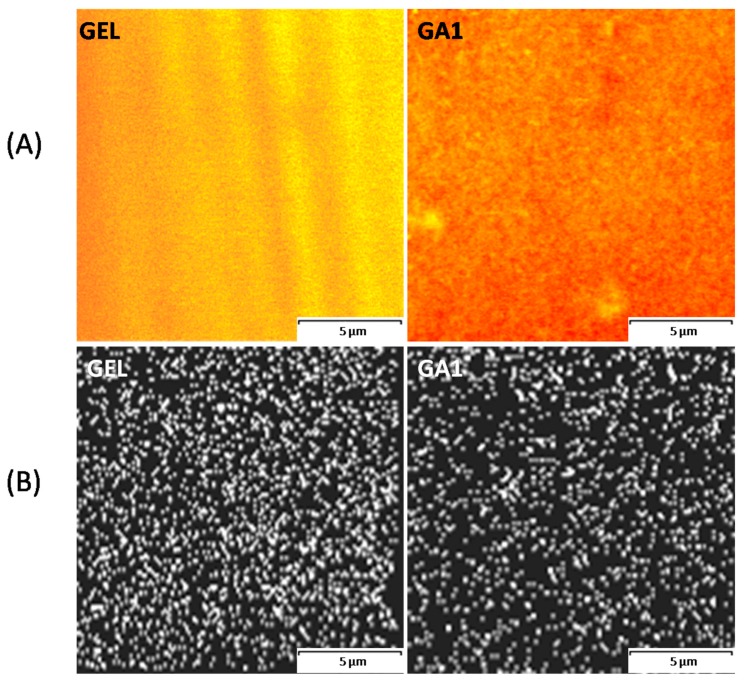
Comparison of the acridine orange distribution (**A**) and nitrogen distribution (**B**) in GEL and GA1 films. Scale bar—5 µm. The stripes on the GEL image (**A**) are the result of the slightly uneven thickness of the investigated film.

The CLSM investigation was conducted on two film types deemed to have the largest structural difference, namely GEL (non-modified film) and GA1 (with the highest Aquacoat content). The microscopic images are shown in Figure 1A. Images were taken after staining the films with acridine orange, which binds to the aminoacids in gelatin. The CSLM images show that non-modified gelatin film is stained yellow to orange, which indicates acridine orange bound to gelatin. The CSLM image of the GA1 sample shows areas of acridine orange bound to protein (stained yellow) dispersed in the darker area (colored red), to which acridine orange was not bound. This indicates the occurrence of distinct CAP and gelatin phases. However, the resolution obtained did not allow for a deeper insight into the film structures. Due to the fact that the gelatin and CAP domains are distinct, it is unlikely for a chemical interaction to occur between these ingredients, while coacervation is more probable. Even though Figure 1A. neither confirms nor rules out such an effect, the low pH of the film-forming mass (in the case of GA1 the pH was 4.5) could promote the coacervation process, since at this pH the gelatin has a partially positive charge (the pI of gelatin is 5.4) and CAP is negatively charged. Such an effect was already described for different polymers [22,23,24,25]. Considering that separation in microdomains is to be expected, as it is generally between two non-interacting polymers, the mixing conditions needed to produce dispersion should be carefully controlled. The formation of a homogenous mass was possible only under a controlled temperature (70–80 °C) and with constant stirring at a slow rate (40 rpm). When the mixing was interrupted before the complete dissolution of the gelatin, a phase separation occurred with the formation of large agglomerates of CAP that were very hard to re-disperse.

Figure 1B presents the nitrogen distribution within samples measured with EDX. The lower density of the nitrogen signals (white spots) in the GA1 film confirms the occurrence of larger areas that are low in nitrogen, which can be identified as CAP domains. However, the nitrogen distribution measurement does not allow for any further conclusions about the internal structure of the modified films.

**Figure 2:**
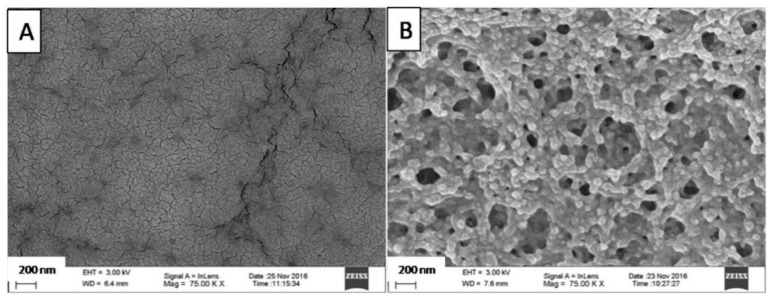
Modified filmsGA1 (30% Aquacoat) before (**A**) and after 3 h in acid (**B**). Scale bar—200 nm.

**Figure 3:**
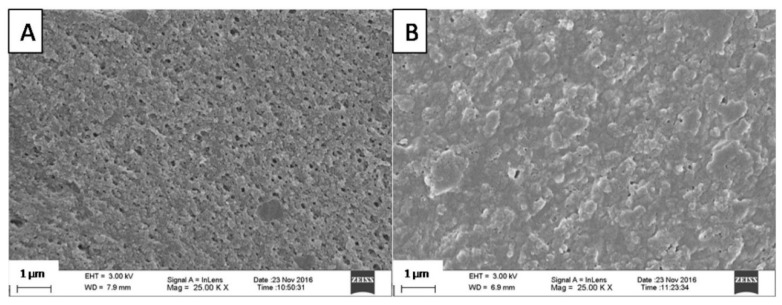
Pore formation during acid treatment of the films: GA2 without carrageenan (**A**) and GA3containing 1.5% carrageenan (**B**). Scale bar—1 µm.

In an attempt to reveal the behavior of the investigated gastro-resistant film samples under the influence of a low pH, the films were exposed to 0.1 M HCl at 37 °C for 3 h. The 0.1 M HCl was selected as a simple acidic solution, which is generally accepted to simulate gastric fluid in vitro. The model makes it possible to investigate the films under a fasted-state strong acidic environment, without considering, however, the higher pH that often occurs in vivo. The freeze-dried acid-treated samples were observed using a SEM and the resulting images are shown in Figure 2 and Figure 3.

The SEM image presented in Figure 2A demonstrates the smooth surface of the GA1 film, without any visible pores. The microstructure of the film is clearly different after contact with 0.1 M HCl (Figure 2B). The acid treatment resulted in a structure resembling a network of agglomerated particles with a considerable porosity visible.

Figure 2B clearly demonstrates that the rather spherical particles of CAP form a well-organized network. Originally, the latex dispersion was emulsion-type, so the particles forming the network are mainly spherical. Since the minimum film-forming temperature of Aquacoat CPD is reported to be approx. 30 °C [26], it can be expected that coalescence of the polymer will occur as the gelatin-CAP mass is heated to 80 °C, thus significantly above this temperature. In any case, full coalescence was not achieved due to the presence of gelatin gel domains, which limited the contact between the latex particles. This resulted in a porous structure that was revealed after dissolving the gelatin domains in 0.1 M HCl.

According to the solubility test conducted (Table 2), the dry mass of the membrane residues after acid treatment is consistent with the initial amount of CAP in the GA1 and GA2 samples. This indicates that during the acid treatment soluble components, i.e., gelatin and glycerol, were washed out from a sample completely, and it could be concluded that the agglomerated structures visible in the SEM images are CAP.

In contrast, the dry residues of the GA3 films (with 1.5% of carrageenan) do not present a clear correlation with the initial CAP content of the films. One may conclude that the addition of carrageenan has a dissolution-hindering effect, which can be attributed to the formation of a polyelectrolyte complex between carrageenan and gelatin [27].

Very surprisingly, the GA4 films containing only 10% Aquacoat did not show a good correlation of the residual mass and initial CAP content. The higher mass of the film remaining after the acid treatment indicated that, besides CAP, around 7% of the gelatin was not dissolved. However, the resulting structure did not contribute to better barrier properties as shown later in Table 3 (see Section 3.2). As a result, the increased residual mass after the acid treatment cannot be explained by gelatin crosslinking. It could also be mentioned that the SEM photos for these samples under greater magnification do not provide any additional data; this is due to sample charging during the investigation (resulting in the blurring of the images). A similar residual mass was observed when films containing 5% Aquacoat were prepared and investigated (data not shown).

Carrageenans are soluble at both a low and high pH, but their dissolution rate depends on the ionic strength of the medium. Nevertheless, the addition of carrageenan inhibited the pore formation effect in GA films, a feature which corresponds well to the permeability test results, as presented further on in this article. Figure 3 shows the differences between the structures of the acid-treated samples. After this treatment, the GA film developed a visible porosity (Figure 3A), while in the GA film containing carrageenan the pore formation was reduced (Figure 3B). The reduction in porosity with the presence of a small amount of carrageenan appears to correspond with the large undissolved fraction in 0.1 M HCl (Table 2), which can be attributed to the well-known formation of the polyelectrolyte complex between gelatin and carrageenan [27,28]. However, such an interaction is undetectable using an IR spectroscopy, as appears in the present study (see Section 3.3). The DSC thermographs of the films prepared with gelatin and carrageenan only revealed a large shift in the endothermic peak of gelatin (from 111.3 °C to 133.1 °C). However, this was not seen in the GA2 and GA3 films (data not shown), which can be explained by the masking effect of the glycerol and CAP present in these films.

**Figure 4:**
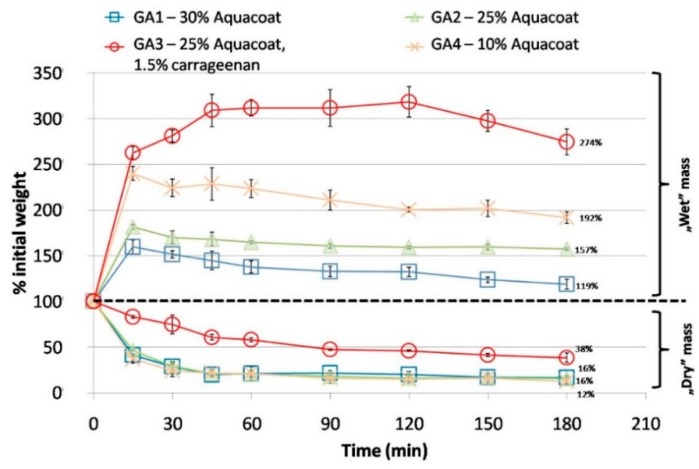
Changes in the weight of the gelatin—Aquacoat films during acid treatment: wet and dry mass.

The swelling test conducted (Figure 4) revealed that the samples containing carrageenan after being submersed in HCl swell noticeably more than carrageenan-free films. This can be explained by the formation of a highly viscous carrageenan-gelatin gel within CAP network structures, which may provide the film with enhanced barrier properties. Moreover, membranes containing carrageenan organoleptically observed appeared to be more mechanically resistant during the test. This could be an important feature in the gastro-resistant performance of the capsules prepared using these compositions.

The data in Figure 4 shows that, depending on the Aquacoat content in the films, different swelling behavior was observed, with a less intensive effect being detected if the fraction of gastro-resistant polymer increased. During the swelling test, after an initial increase in the mass of the samples, a subsequent reduction in the weight was observed, which can be explained by the gradual rinsing out of the soluble components from the films. The process was delayed, however, in films containing carrageenan, as the weight of the sample was reduced only after 150 min.

The dry residue of the films after the test was analyzed as a measure of the erosion. This was independent of the concentration of the Aquacoat added. However, the effect of carrageenan was again observed: From the larger dry residue, one can conclude that in the acidic environment the structure of GA film containing carrageenan was less eroded.

### 3.2. Permeability

The permeability test was conducted for the films containing 30, 25 and 10% Aquacoat (GA1, GA2 and GA4, respectively), as well as for the film sample additionally modified with ί-carrageenan (GA3). Moreover, the samples of pure Aquacoat CPD films were prepared through casting (cured at 55 °C for 24 h, final thickness 600 µm) and their permeability was also measured.

The linear increase in radioactivity in the acceptor fluid was determined in the case of all the investigated films. However, the films with the lowest Aquacoat content (10%-GA4) disintegrated during the test after 100–150 min.

Based on the DPM values measured, the concentration of radio-labeled water was calculated. In relation to the linear increase in radioactivity in the acceptor chamber, the Fickian diffusion equation was applied (see Section 2.8).

**Figure 5:**
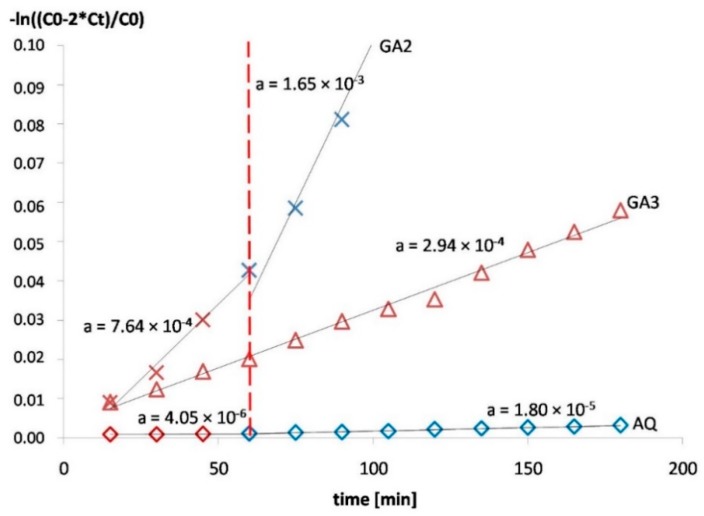
Permeation rate of T_2_O through GA2 (25% Aquacoat), GA3 (25% Aquacoat, 1.5% carrageenan) and AQ (pure Aquacoat CPD) films. The slopes for different experiments are fitted and the “a” values for the slopes are indicated in the graph.

After plotting the concentration gradient logarithm against time (Figure 5) it became visible that two stages can be distinguished in the diffusion process, with a slower diffusion of tritium-labeled water within the first 60 min (Table 3). This was not the case, however, for the composition additionally modified with carrageenan (GA3).

In the GA2 films, the increase in permeability can be related to the equilibration phase seen in the swelling and mass loss test. As presented in Figure 4 earlier, one can also see that the decrease in mass levels out after about 60 min, which probably corresponds to the time needed to create the final porous structure. Therefore, after 60 min all the soluble ingredients are “rinsed out” from the membranes, the pore formation reaches the maximum extent, and the diffusion of the permeant becomes higher. However, the two-stage process was also observed for the films made with pure Aquacoat CPD (AQ), albeit to a far lesser extent (Figure 5, Table 3). In this case, however, no significant porosity was observed. The difference in apparent permeability before and after 60 min for the AQ film could possibly be explained by the fast washing out of the glycerol and poloxamer, which results in increased T_2_O transport rates at later times.

The two-stage permeability pattern was not observed in the GA3 films (Figure 5, Table 3). In the case of these samples, the presence of an additional gelling agent resulted in a delay in the permeation through such membranes under investigated conditions, by causing the acid-soluble components of the membrane to be dissolved at a slow, constant rate. In this case, it is possible that the second stage of flow through the membrane becomes visible after a longer time. Therefore, the mechanism of water transport through the GA3 films is based on diffusion through a homogenous hydrogel layer more than through a porous structure. This appears to correlate well with the large mass increase in the swelling test reported above (Figure 4).

### 3.3. ATR-FTIR

The ATR analysis was conducted for the dry samples of both modified and non-modified films. Each investigated sample was scanned on both sides of the film: the topside (in contact with air during the preparation step) and the bottom side (in contact with the glass plate). The IR spectra showed no difference between the scans of each side, which indicates that no surface phase separation took place in the investigated compositions.

**Figure 6:**
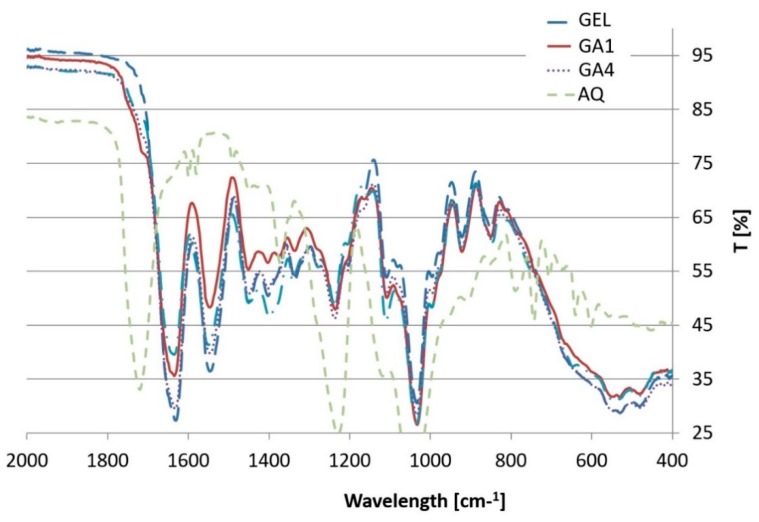
FTIR spectra of bovine-gelatin based formulations (GEL, GA1, GA4,) and spectrum of the AQ film.

The overlaid spectra of the formulations are shown in Figure 6. The results show no evidence of chemical interactions between the ingredients. However, some subtle differences between certain formulations need to be considered.

The spectra of the films based on gelatin contain peaks that are characteristic of gelatins at all wavenumber values [7,29,30].

According to literature data, a peak shift in the spectra of modified films could suggest a chemical interaction between the ingredients, as in the case of the gelatin films reinforced with chitosan nanoparticles, where a shift in amide A and amide II towards higher wavenumbers was reported, indicating possible hydrogen bonding between –OH, –COOH and NH groups of gelatin and –OH and NH_2_ groups of chitosan [31]. In our study, only slight shifts in peak wavenumbers were observed, which could indicate the creation of hydrogen bonds between the mixed polymers. The peak at 1631 cm^−1^ in the non-modified gelatin film (GEL) spectrum appears to shift towards a higher wavenumber to 1635 cm^−1^ in the modified films (GA1, GA2, GA3) and to 1633 cm^−1^ in the GA4 composition. However, the difference of 4 cm^−1^ could have been caused by the fact that the pH of film-forming masses was slightly below the isoelectric point of the gelatin (pH of compositions ca. 4.5, IP of gelatin 4.7–5.6), thus the amide carbonyl groups were more likely to accept a hydrogen bond. The hydrogen donor in this situation could be the –NH group of another gelatin side chain. In this scenario, the hydrogen bond created could instead be attributed to the helical structure of the gelatin, which indicates an interaction between the film components. Moreover, no new peaks were observed, which strongly suggests that no interaction of a chemical nature occurred between the constituents of the modified gelatin films.

**Figure 7:**
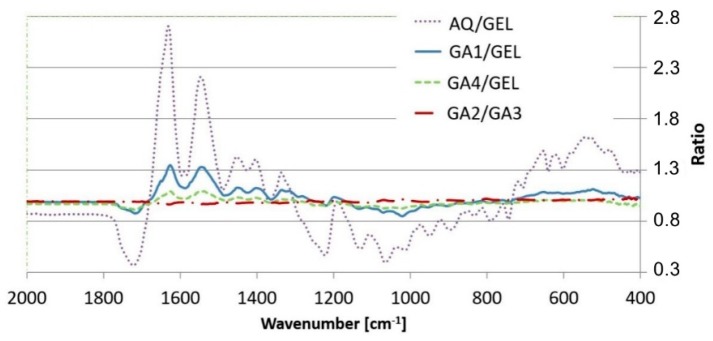
Peak intensity ratios of the films.

The spectra were compared by calculating the peak intensity ratio [32]. The peak intensity ratio data correlates well with the Aquacoat content in the modified film samples and changes along with increasing content of the Aquacoat in the compositions (Figure 7). At 1718 cm^−1^, an intensified signal of carbonyl most likely comes from the acid group. The difference at 1626 cm^−1^ and 1540 cm^−1^ may represent weaker signals, respectively, from bending vibrations of amide I and II N–H bands of the gelatin chains.

Despite changing the properties of the films, the addition of iota-carrageenan (1.5%) did not alter the IR spectrum. The deviation of the peak intensity ratios, from one in this case, seems to be negligible. Although the sulfate groups of carrageenan make it possible to form an electrostatic interaction with positively charged groups of gelatin [33], the IR spectra obtained do not provide any evidence of such an interaction. It is probable that because of the low concentration of carrageenan (1.5%) or the presence of CAP, the interaction occurs but is not detectable with FTIR, similarly to its not being shown by DSC, as mentioned above. This outcome was reported previously [27].

## 4. Conclusions

The present study provided an insight into processes leading to the formation of gastro-resistant gelatin-based films prepared with the addition of a commercially available CAP dispersion, as well as into water permeation mechanisms through the films. The results show that gelatin films modified with an aqueous dispersion of CAP maintain their integrity at an acidic pH (1.2), yet the creation of significant pores was observed. Despite their porous structure, the films retained barrier properties in an acidic environment. An increase in water permeability after 60 min of the test was observed, but this effect can be noticeably hindered by the addition of ί-carrageenan to the film composition.

The analyses performed do not provide any proof of chemical interactions between the components of the investigated films. The microscopic images suggest the coalescence of CAP particles and the formation of a network structure. The addition of ί-carrageenan to the samples reduced the formation of the porous structure and noticeably diminished the dissolution of modified films, thus increased dry residue after 3h of soaking in 0.1M HCl was determined. This suggests that besides CAP, a part of the materials in the carrageenan-modified films resisted the acid treatment.

## Figures and Tables

**Table 1:** Compositions of modified-and reference films.

Symbol	% Aquacoat CPD *	Composition of Dry Film (%)			
		Gelatin	Aquacoat CPD	Glycerol	ί-Carrageenan
GA1	30%	48.1	20.6	31.3	-
GA2	25%	51.5	17.2	31.3	-
GA3	25%	50.0	17.2	31.3	1.5
GA4	10%	61.8	6.9	31.3	-
GEL	0%	68.7	-	31.3	-
AQ	100%	-	100.0	-	-

* %m/m in the Aquacoat—gelatin mixture calculated on a dry basis (the Aquacoat fraction relative to the film-forming components of the film).

**Table 2:** Composition of the dry films (%m/m) and dry residues recovered after 3 h in 0.1 M HCl.

Sample	Content (% m/m) in the Investigated Films			Residue (% of the Initial Mass) *
	Aquacoat CPD Content		Iota-Carrageenan	
	Total	CAP		
GA1	20.6%	15.8%	-	16.4 ± 2.1%
GA2	17.2%	13.2%	-	15.5 ± 1.2%
GA3	17.2%	13.2%	1.5%	38.3 ± 5.1%
GA4	6.9%	5.3%	-	12.4 ± 2.0%

* Average ± SD, *n* = 3.

**Table 3:** Permeation of water through the investigated films.

Films			Permeation of Water *		
Sample	Aquacoat % (Based on Total Polymer Content)	Thickness (µm)	Cumulative Amount after 3 h (% of the Total Amount)	Average Permeability (cm^2^/s)	
				15–60 min	60–180 min
GA1	30%	540 ± 17	7.6%	2.65 × 10^−6^	4.02 × 10^−6^
GA2	25%	509 ± 27	10.9%	3.53 × 10^−6^	5.95 × 10^−6^
GA3	25% (+1.5% carrageenan)	622 ± 35	2.3%	1.1 × 10^−6^	
GA4	10%	446 ± 17	n/a	8.19 × 10^−6^	Disintegration after 100–150 min
AQ	100%	600 ± 17	0.15%	1.67 × 10^−8^	7.58 × 10^−8^

* experiments were performed in duplicates with a difference of less than 10% between the results.
